# Choroidal morphologic and vascular features in patients with unilateral idiopathic epiretinal membranes: An optical coherence tomography analysis integrated with assessment of retinal layers

**DOI:** 10.3389/fmed.2022.1083601

**Published:** 2023-01-06

**Authors:** Xinglin Wang, Jiarui Yang, Changguan Wang, Xuemin Li

**Affiliations:** ^1^Department of Ophthalmology, Peking University Third Hospital, Beijing, China; ^2^Beijing Key Laboratory of Restoration of Damaged Ocular Nerve, Beijing, China

**Keywords:** idiopathic epiretinal membrane, enhanced depth imaging optical coherence tomography (EDI-OCT), choroidal vascularity index, choroidal capillary perfusion, choroidal vasculature

## Abstract

**Introduction:**

Integrated analysis of retinal and choroidal morphologic and vascular features is urgently needed to examine whether and how these two elements interact with each other, thus contributing to visual impairment in patients with idiopathic epiretinal membranes (iERMs).

**Methods:**

An observational retrospective study consisting of 181 patients diagnosed with unilateral iERM between August 2019 and July 2022 was carried out at Peking University Third Hospital. All patients underwent a standardized set of ophthalmologic examinations, including EDI-OCT and OCTA scanning, and were subsequently categorized into four stages according to current classification schemes based on their OCT findings. Altogether, 15 qualitative and quantitative parameters of both the retina (full-layer, inner and outer layers) and choroid were identified.

**Results:**

The results revealed variations in the choroidal vascularity index (CVI) among different stages of iERMs (*p* < 0.001) for the first time. Distributions of retinal parameters across four stages of iERMs were validated. Correlation analysis between choroidal and retinal parameters showed that the CVI was associated with both inner and outer retinal morphologic biomarkers. Functional damage to retinal integrity was determined to be a strong contributor to visual acuity reduction in iERMs.

**Discussion:**

This study complemented our present understanding of posterior segment structural and vascular alterations in iERMs.

## Introduction

Idiopathic epiretinal membrane (iERM) is a common macular abnormality in elderly people characterized by aggregation of extracellular matrix and pre-retinal proliferation of myofibroblastic cells. Patients are often asymptomatic at first, but iERMs eventually induce progressive worsening of visual functions, including blurred vision and metamorphopsia (1, 2).

The introduction of current imaging modalities, such as spectral-domain optical coherence tomography (SD-OCT), enhanced depth imaging OCT (EDI-OCT), and optical coherence tomography angiography (OCTA) has enabled us to quantify the microstructure in the retina and choroid in patients with iERMs (3, 4). Previous studies have elucidated the morphologic alterations of different layers in the inner retina, including the ganglion cell layer (GCL) (5–10), inner plexiform layer (IPL) (5–10), inner nuclear layer (INL) (5–11), and ectopic inner foveal layer (EIFL) (12–17), which is defined as a unique inner retinal layer when distortion of the retina is present. The identification of EIFL also facilitated a four-stage iERM classification system which has been widely used and validated recently (13). The microstructure of the outer retina, including ellipsoid zone (EZ) and central bouquet (CB) abnormalities in iERMs was also investigated (14, 18–24). However, the tractional and tangential forces induced by iERM may transmit downward thus impacting the underlying choroid (14). In this regard, observations of retinal architecture alone are insufficient to understand the pathophysiology of iERMs. Besides, the incorporation of both retinal and choroidal parameters is urgently needed to examine whether and how these two elements interact with each other and with patient visual outcomes.

In this study, we aimed to determine the variations in retinal (both inner and outer layers) and choroidal parameters in different stages of iERMs and identify the factors related to best corrected visual acuity (BCVA). The association between retinal and choroidal parameters was also evaluated.

## Materials and methods

### Study design and participants

An observational, retrospective, institutional case series was carried out at Peking University Third Hospital in adherence to the tenets of the Declaration of Helsinki and was approved by the Institutional Review Board of Peking University Third Hospital.

**FIGURE 1:**
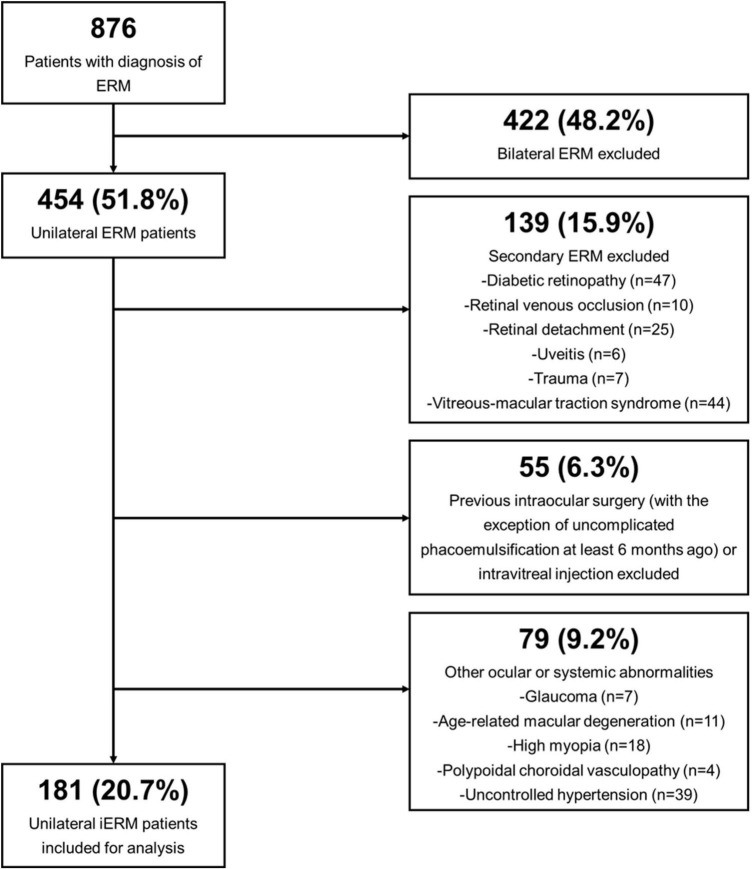
Flow diagram demonstrating the selection process of enrolled unilateral idiopathic epiretinal membrane (iERM) subjects.

Electronic records of patients diagnosed with unilateral iERM between August 1st 2019 and July 31st 2022 according to existing standards (1) were examined. The exclusion criteria were as follows: (1) secondary ERM (2) history of previous intraocular surgery with the exclusion of uncomplicated phacoemulsification at least 6 months ago; (3) presence of other ocular abnormalities that could have interfered with functional and morphologic results, as summarized in Supplementary Table 1; (4) contralateral eye suffering from any form of retinochoroidal diseases. Details of patient enrollment are illustrated in Figure 1.

### Ocular examination

All patients underwent a standardized set of ophthalmologic examinations, including slit-lamp biomicroscopy, dilated fundus examination, EDI-OCT and OCTA scanning, as previously described (25, 26). Best-corrected visual acuity (BCVA) was obtained after autorefraction screening (Canon Autorefractor RK-F1; Canon Inc., Ltd., Tochigiken, Japan) and manifest refraction in the Snellen fraction and was converted into the logarithm of the minimum angle of resolution (logMAR) for statistical analysis. Intraocular pressure (Auto Non-contact Tonometer, NT-3000; Nidek, Gamagori, Japan) and biometric measurements (Non-contact Zeiss IOLMaster-700, Carl Zeiss Meditec AG, Jena, Germany) were also assessed. Both eyes of the included subjects were selected for examination and subsequent analysis.

### Acquisition of retinal and choroidal parameters and grading of iERMs

All retinal and choroidal structural parameters were obtained using Heidelberg Spectralis SD-OCT (Heidelberg Engineering, Heidelberg, Germany) in EDI-OCT mode (cross-line scan spanning 30°) and OCTA (Optovue Inc., Freemont, California, USA). All OCT measurements were taken in a 2 mm-wide area centered at the fovea to match the 1 mm-radius circular calculation area in OCTA, which is elucidated below. Choroidal vascularity index (CVI) was defined as the ratio of choroidal luminal area (LA) and total choroidal area (TCA) based on post-binarization OCT images. Subfoveal choroidal thickness (SFCT), TCA, LA, choroidal stromal area (SA), and CVI were all measured and calculated automatically by a software which we developed as previously described (25). Images with poor identification of the choroidal-scleral interface or scanning quality below 6/10 after two rescan attempts were excluded. To avoid inaccurate segmentation of the retinal layers in advanced iERMs where disorganization of the retina is commonly present, two masked ophthalmologists were assigned to manually evaluate central retinal thickness (CRT) and the thickness of retinal GCL, IPL, INL, EIFL, and photoreceptor outer segment (PROS), with intraclass correlation coefficients (ICC) of 0.961 (CRT), 0.954 (GCL + IPL), 0.966 (INL), 0.928 (EIFL), and 0.957 (PROS), and the mean value of their measurements were selected for subsequent analysis. Boundaries of each retinal layer evaluated are embedded in Supplementary Table 2, and an example of measurements of retinal thickness is illustrated in Supplementary Figure 1. EZ disruption and CB abnormalities were assessed according to a previous classification scheme (14). Choroidal capillary perfusion (CCP), as a reference for choroidal capillary blood flow, was quantitatively evaluated on OCTA in angio-retina scanning mode (840 nm, 70,000 A-scans/s, 3 mm × 3 mm) (27, 28).

Furthermore, iERMs were classified into four stages by the SD-OCT classification system proposed by Govetto et al. (13) depending on the preservation (Stage 1) or absence (Stage 2) of a foveal pit, presence of ectopic inner foveal layers (Stage 3) and disorganization of the retinal layers (Stage 4).

### Statistical analysis

Statistical analysis was performed using SPSS version 20.0 (SPSS, Inc., Chicago, IL), and all values are represented as mean ± standard deviation unless indicated otherwise. The normality of the distributions of all variables was assessed using analytical methods (Shapiro-Wilk or Kolmogorov-Smirov tests). Independent *t*-tests or Mann-Whitney *U*-tests were used to compare continuous variables between two groups of iERMs. One-way ANOVA, Kruskal-Wallis or Welch tests were selected to compare continuous variables among three or more groups. Pearson Chi-Square test or Fisher’s exact test was used to compare proportions among the study population. The correlations between retinal and choroidal parameters and between all parameters and BCVA were calculated by Spearman rank correlation or Kendall’s tau-b correlation analysis. Multiple regression analysis was performed to determine the independent predictors of BCVA after a collinearity check. A two-sided *p*-value less than 0.05 was considered to be statistically significant.

## Results

A total of 181 patients suffering from unilateral iERM were included in the study, consisting of 94 men and 87 women from 53 to 81 years old (mean age: 66.73 ± 6.44 years). The average logMAR BCVA was 0.36 ± 0.28 (20/39) in affected eyes and 0.10 ± 0.08 (20/25) in contralateral eyes. Based on OCT imaging findings, the iERM status of all patients was classified into stage 1 (54 eyes, 29.8%), stage 2 (51 eyes, 28.2%), stage 3 (45 eyes, 24.9%), and stage 4 (31 eyes, 17.1%). No significant difference in age, gender distribution, axial length or refractive error was identified among all groups (Table 1).

**TABLE 1:** Demographics and ocular characteristics in different stages of idiopathic epiretinal membrane and fellow eyes.

	Stage 1 (*n* = 54)	Stage 2 (*n* = 51)	Stage 3 (*n* = 45)	Stage 4 (*n* = 31)	*P*-value	Fellow eyes (*n* = 181)
Age (years)	65.94 ± 4.24	66.98 ± 6.99	66.24 ± 7.47	68.42 ± 7.01	0.331 ^a^	/
Gender (Male/Female)	32/22	27/24	19/26	16/15	0.410 ^b^	/
Presence of diabetes	6/54	8/51	7/45	3/31	0.813 ^e^	/
Axial length (mm)	24.25 ± 1.21	24.52 ± 1.09	24.61 ± 1.33	24.58 ± 1.46	0.772 ^a^	24.39 ± 1.78
Refractive error (D)	−0.49 ± 2.56	−0.26 ± 2.94	−0.17 ± 2.74	−0.44 ± 3.14	0.549 ^a^	−0.24 ± 3.22
**Retinal parameters**						
CRT (μm)	292.87 ± 30.24	415.90 ± 34.68	518.00 ± 31.21	636.77 ± 49.01	<0.001^a^	253.12 ± 20.84***
GCL + IPL (μm)	102.25 ± 18.03	153.80 ± 23.94	173.86 ± 16.37	/	<0.001^c^	97.43 ± 10.26***
INL (μm)	46.82 ± 10.94	53.39 ± 10.81	56.80 ± 10.10	/	<0.001^d^	44.55 ± 5.19***
GCL + IPL + INL (μm)	149.08 ± 27.35	207.19 ± 31.94	230.66 ± 24.95	/	<0.001^d^	138.96 ± 10.85***
EIFL (μm)	/	/	186.07 ± 51.94	/		/
PROS length (μm)	61.78 ± 3.27	67.60 ± 11.23	69.47 ± 14.07	63.06 ± 6.13	0.097 ^a^	63.80 ± 5.50*
EZ disruption	2/54 (3.7%)	9/51 (17.6%)	12/45 (26.7%)	14/31 (45.2%)	<0.001^b^	/
Central bouquet abnormalities	3/54 (5.6%)	13/51 (25.5%)	10/45 (22.2%)	3/31 (9.7%)	0.011^e^	
Cotton ball sign	3/54	7/51	2/45	0/31		/
Foveolar detachment	0/54	4/51	5/45	3/31		/
Vitelliform lesion	0/54	2/51	3/45	0/31		/
Presence of CME	0/54 (0%)	7/51 (13.7%)	9/45 (20.0%)	11/31 (35.5%)	<0.001^b^	/
**Choroidal parameters**						
SFCT (μm)	232.30 ± 25.29	226.88 ± 38.95	233.50 ± 43.75	238.62 ± 60.04	0.471^a^	233.16 ± 43.40
TCA (mm^2^)	0.4435 ± 0.0575	0.4481 ± 0.0651	0.4621 ± 0.0535	0.4710 ± 0.0656	0.423 ^a^	0.4614 ± 0.0822
LA (mm^2^)	0.2822 ± 0.0370	0.2893 ± 0.0423	0.3015 ± 0.0351	0.3086 ± 0.0429	0.045 ^a^	0.2922 ± 0.0521
SA (mm^2^)	0.1613 ± 0.0205	0.1588 ± 0.0230	0.1606 ± 0.0188	0.1624 ± 0.0230	0.917 ^a^	0.1693 ± 0.0302***
CVI	0.6362 ± 0.0035	0.6455 ± 0.0058	0.6524 ± 0.0060	0.6553 ± 0.0062	<0.001^a^	0.6332 ± 0.0038***
CCP (%)	57.37 ± 3.41	56.99 ± 5.41	55.58 ± 5.26	56.78 ± 5.81	0.406 ^a^	63.45 ± 4.35***
BCVA (logMAR)	0.10 ± 0.07	0.29 ± 0.12	0.46 ± 0.18	0.79 ± 0.25	<0.001^a^	0.10 ± 0.08***

CRT, central retinal thickness; CME, cystoid macular edema; GCL + IPL, ganglion cell layer + inner plexiform layer; INL, inner nuclear layer; EIFL, ectopic inner foveal layer; EZ, ellipsoid zone; PROS, photoreceptor outer segment; SFCT, subfoveal choroidal thickness; TCA, total choroidal area; LA, luminal area; SA, stromal area; CVI, choroidal vascularity index; CCP, choroidal capillary perfusion; BCVA, best corrected visual acuity. Asterisks represent the significance comparing values in iERMs and their contralateral eyes. *p < 0.05; ***p < 0.001.

^a^Kruskal-Wallis test.

^b^Pearson Chi-Square test.

^c^Welch test.

^d^One-way ANOVA test.

^e^Fisher’s exact test.

### Variations in retinal and choroidal parameters in different stages of iERMs

The anatomical and functional parameters in different stages of iERMs and fellow eyes are shown in Table 1. The overall retina and inner retinal layers, including the GCL, IPL, and INL, thickened in higher stages of iERMs (*p* < 0.001, Supplementary Figure 1), with a higher occurrence of complicated CME (*p* < 0.001). Structural disturbance in the outer retina varied significantly among the four groups (*p* < 0.001 for EZ disruption, *p* = 0.011 for CB abnormalities). The subtype of CB abnormalities was also significantly associated with iERM stages (*p* = 0.030), where stage 2 iERMs had the most cotton ball signs (13.7%) and stage 3 iERMs had the most foveolar detachments (11.1%). The PROS length in iERMs was higher than that in the control group (65.55 ± 10.16 vs. 63.80 ± 5.50, *p* = 0.047), but was comparable among the four stages of iERMs (*p* = 0.097). CVI, the only choroidal parameter which was significantly different among the four groups, exhibited an increasing pattern as iERM advanced into higher stages (*p* < 0.001).

### Associations of retinal and choroidal parameters in iERMs

**FIGURE 2:**
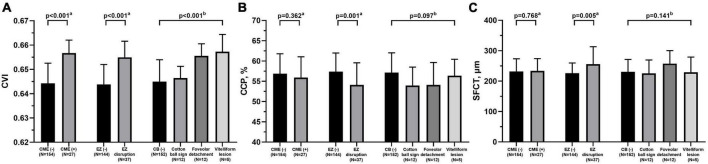
Comparisons of choroidal parameters including CVI **(A)**, CCP **(B)**, and SFCT **(C)** between eyes with different retinal features under idiopathic epiretinal membrane conditions. CVI, choroidal vascularity index; CCP, choroidal capillary perfusion; SFCT, subfoveal choroidal thickness; CME, cystoid macular edema; EZ, ellipsoid zone; CB, central bouquet abnormality. ^a^Mann-Whitney test. ^b^Kruskal-Wallis test.

The correlation analysis between retinal and choroidal parameters in eyes with iERMs revealed that a higher CVI correlated with a thicker CRT, GCL + IPL, INL, GCL + IPL + INL, and PROS (Table 2). As shown in Figure 2, eyes accompanied with CME had a higher CVI (*p* < 0.001) and statistically equivalent CCP (*p* = 0.362) and SFCT (*p* = 0.768) than uncomplicated iERM eyes. All choroidal parameters including CVI (*p* < 0.001), CCP (*p* = 0.001), and SFCT (*p* = 0.005) varied between eyes with and without EZ disruption. Additionally, only the CVI out of all choroidal parameters was affected in terms of CB status (*p* < 0.001, *p* = 0.097, *p* = 0.141 for CVI, CCP, and SFCT, respectively; detailed data are embedded in Table 3).

**TABLE 2:** The association between retinal and choroidal parameters in eyes with epiretinal membranes (*N* = 181).

	CRT, μm	GCL + IPL, μm^a^	INL, μm^a^	GCL + IPL + INL, μm^a^	EIFL, μm^b^	PROS, μm
**CVI**	*r* = 0.792* *p* < 0.001	*r* = 0.690* *p* < 0.001	*r* = 0.272* *p* = 0.001	*r* = 0.655* *p* < 0.001	*r* = 0.197 *p* = 0.195	*r* = 0.343* *p* < 0.001
**CCP,%**	*r* = −0.089 *p* = 0.235	*r* = −0.119 *p* = 0.146	*r* = −0.113 *p* = 0.169	*r* = −0.124 *p* = 0.130	*r* = 0.012 *p* = 0.938	*r* = −0.113 *p* = 0.181
**SFCT, μ m**	*r* = −0.072 *p* = 0.335	*r* = −0.133 *p* = 0.104	*r* = −0.031 *p* = 0.702	*r* = −0.120 *p* = 0.145	*r* = −0.086 *p* = 0.573	*r* = −0.067 *p* = 0.369

CRT, central retinal thickness; GCL + IPL, ganglion cell layer + inner plexiform layer; INL, inner nuclear layer; EIFL, ectopic inner foveal layer; PROS, photoreceptor outer segment; BCVA, best corrected visual acuity; CVI, choroidal vascularity index; CCP, choroidal capillary perfusion; SFCT, subfoveal choroidal thickness.

*Values with statistical significance were highlighted with asterisks.

^a^*N* = 150 (stage 1–3 epiretinal membranes with identifiable retinal layers).

^b^*N* = 45 (stage 3 epiretinal membranes).

**TABLE 3:** Distributions of choroidal parameters including CVI, CCP, and SFCT in iERMs eyes in terms of different structural retinal status.

	CVI	CCP	SFCT
CME (−) (*N* = 154)	0.6443 ± 0.0083	56.86 ± 4.91	231.87 ± 41.47
CME (+) (*N* = 27)	0.6567 ± 0.0053	55.90 ± 5.14	233.78 ± 40.05
*P*-value^a^	<0.001	0.362	0.768
EZ (−) (*N* = 144)	0.6438 ± 0.0082	57.40 ± 4.58	226.09 ± 33.42
EZ disruption (*N* = 37)	0.6550 ± 0.0066	54.08 ± 5.48	255.74 ± 57.57
*P*-value^a^	< 0.001	0.001	0.005
CB (−) (*N* = 152)	0.6450 ± 0.0090	57.15 ± 4.86	230.77 ± 40.28
CB (+) (*N* = 29)	0.6521 ± 0.0070	54.44 ± 4.81	239.41 ± 45.54
*P*-value^a^	<0.001	0.015	0.410
Cotton ball sign (*N* = 12)	0.6465 ± 0.0048	53.96 ± 4.53	225.39 ± 44.17
Foveolar detachment (*N* = 12)	0.6556 ± 0.0050	54.12 ± 5.52	257.69 ± 42.40
Vitelliform lesion (*N* = 5)	0.6573 ± 0.0071	56.37 ± 4.04	229.21 ± 49.88
*P*-value^b^	<0.001	0.097	0.141

CVI, choroidal vascularity index; CCP, choroidal capillary perfusion; SFCT, subfoveal choroidal thickness; CME, cystoid macular edema; EZ, ellipsoid zone; CB, central bouquet abnormality.

^a^Mann-Whitney test.

^b^Kruskal-Wallis test among three CB (+) groups and CB (−) group.

### Predictive variables affecting BCVA in iERMs

**FIGURE 3:**
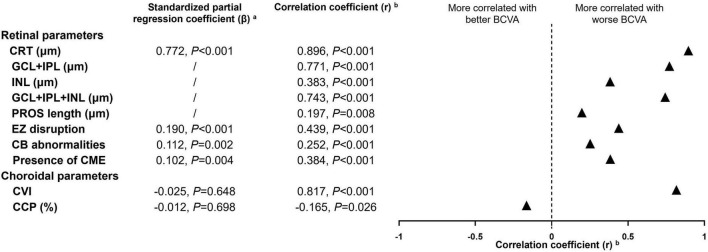
Multiple regression and correlation analysis to determine the OCT morphologic factors related to best-corrected visual acuity in idiopathic epiretinal membranes. CRT, central retinal thickness; CME, cystoid macular edema; GCL + IPL, ganglion cell layer + inner plexiform layer; INL, inner nuclear layer; EZ, ellipsoid zone; CB, central bouquet; PROS, photoreceptor outer segment; CVI, choroidal vascularity index; CCP, choroidal capillary perfusion; BCVA, best corrected visual acuity. ^a^OCT parameters with statistically solid and integral values across four stages of idiopathic epiretinal membranes were included in multiple regression analysis, which ruled out thickness of specific retinal layer for the disrupted retinal structure in higher stages of iERMs. Subfoveal choroidal thickness and choroidal area parameters were also excluded for their comparable value among four groups and lack of correlation to BCVA. ^b^Spearman correlation analysis was used for continuous variables and Kendall’s tau-b correlation analysis was used for categorical variables.

Figure 3 demonstrates the correlation of BCVA and various OCT morphologic biomarkers in iERMs. Among all retinal and choroidal parameters, CRT, CVI, and GCL + IPL thickness had the strongest correlation with BCVA (*r* = 0.896, 0.817, 0.771, respectively. All *p* < 0.001), followed by GCL + IPL + INL thickness (*r* = 0.743, *p* < 0.001), EZ disruption (*r* = 0.439, *p* < 0.001), INL thickness (*r* = 0.383, *p* < 0.001), CB abnormalities (*r* = 0.252, *p* < 0.001), PROS length (*r* = 0.197, *p* = 0.008), and CCP (*r* = −0.165, *p* = 0.026). SFCT was found to be statistically irrelevant to BCVA (*r* = −0.006, *p* = 0.937). Subsequently, parameters with statistically solid and integral values across four stages of iERMs were included in multiple regression analysis, which ruled out GCL and IPL for the disrupted retinal structure in higher stages of iERMs. SFCT and choroidal area parameters were also excluded for their comparable values among the four groups and lack of correlation with BCVA. The regression model (*R*^2^ = 0.843, adjusted *R*^2^ = 0.837) showed that the predictive variables for BCVA were CRT (standardized partial regression coefficient β = 0.772, *p* < 0.001), presence of CME (β = 0.102, *p* = 0.004), EZ disruption (β = 0.190, *p* < 0.001), and CB abnormalities (β = 0.112, *p* = 0.002). None of the choroidal parameters exhibited predictive value in terms of BCVA.

## Discussion

Much evidence regarding the posterior segment microstructure using non-invasive OCT and OCTA has emerged in recent years, with attempts to identify predictive and prognostic factors of visual functioning in iERMs (6, 8, 9, 13–15, 17–22, 29). However, these investigations mostly focused on retinal features, and even with a handful of studies revealing the potential role of the choroid in iERMs (28, 30–33), all-around incorporation of OCT and OCTA biomarkers was still insufficient to achieve. Furthermore, fewer studies have been updated according to the ERM classification and staging scheme (1, 13). To the best of our knowledge, this is the first study to comprehensively quantify the variations in retinal morphology at the full-layer, inner retina and outer retina levels, together with choroidal morphological and vascular parameters in all four stages of iERMs.

Here, our study confirmed that CRT and the presence of CME were elevated in higher stages of iERMs, which has been widely described before (12–14, 16, 34, 35). The microstructure of the inner retina has also been quantitatively evaluated by previous investigators, but the conclusions were somewhat conflicting regarding some specific inner retinal parameters, including the thickness of the GCL + IPL and INL. In some cases, GCL + IPL was thinner in eyes with iERM than in healthy controls (6, 7), while others discovered the opposite results (5, 8–10). This is reasonable considering that the auto segmentation and assessment of the inner retina through the built-in ganglion cell analysis (GCA) algorithm on OCT usually fails when severe retinal layer distortion is present in cases of iERMs, which makes the identification of the GCL, IPL, and INL mostly dependent on manual measurements. Besides, the fact that patients in these studies were not classified or graded according to a unanimous staging scheme may also contribute to these contrary results. In the present study, we found that the thickness of both the GCL + IPL and INL varied among different stages of iERMs, with stage 3 iERMs being the thickest followed by stage 2 and stage 1 iERMs (stage 4 iERMs were excluded for their inner retinal distortion), and all iERMs had thicker GCL + IPL and INL than the control group, which was in accordance with most previous studies (5, 8–10). Although still relying on manual measurements, our study provides the first evidence to distinguish variations in inner retinal morphology in different stages of iERMs.

The tangential reaction caused by ERM, which induced lateral displacement of the inner retina and thickened the GCL, IPL, and INL within (8, 14, 36), also affected the outer retina in our subjects. The results here demonstrated that EZ disruptions were presented more frequently in higher stages of iERMs, and subtypes of CB abnormalities were also significantly associated with iERM stages, which is in line with a previous study by Govetto et al. (14). PROS length was longitudinally analyzed before and was found to be higher preoperatively and decreased after membrane peeling in iERMs (21, 37), but comparisons among different stages of iERMs and with healthy control eyes are still lacking. Our results here showed that iERMs had a higher PROS length than controls and that PROS length was not associated with iERM stages.

A few studies dug into choroidal morphologic and vascular features using SFCT, CVI, or CCP separately for observations of iERM patients (32, 38–41), but their investigation was neither integrated in terms of choroidal parameters nor did they distinguish the different stages of iERMs. Our systematic analysis of the choroid showed that SFCT was unaffected by the presence or severity of iERM. This was also validated by the comparable values of TCA, LA, and SA in all groups, suggesting that no expansion or shrinking of the choroid total volume was observed in iERMs. However, the choroid appeared more vascularized in higher stages of iERMs on the full-layer level (*P* < 0.001 for CVI among all groups), which complemented the preliminary findings of a previous study where iERMs had a higher CVI than healthy eyes (39). In accordance with a recent study in which eyes with iERM had lower CCP than contralateral eyes (31), similar results were identified here [affected eyes vs. healthy eyes (%), 56.72 ± 4.94 vs. 63.45 ± 4.35, *P* < 0.001], but subsequent subgroup analysis revealed that CCP was independent of iERM stages.

All main choroidal parameters including SFCT, CVI, and CCP showed variations in terms of the presence of EZ disruption. Without longitudinal follow-up on iERM development, we cannot say for certain whether the altered choroidal morphology and vascularity affected EZ continuity or vice versa. Based on the fact that the densely vascularized choroid, especially its Haller’s layer, provides nutrient and oxygen exchange with the outer retina (4, 42), we speculate that the tangential traction caused by iERMs not only mechanically stretches the EZ band (or all outer retina), but also disturbs choroidal function, which results in EZ injury. CVI was found to be correlated with all retinal parameters except the thickness of EIFL, indicating that the previously neglected choroid may actually play a crucial role in the development of retinal distortion under iERM status.

However, it is noteworthy that even with significant correlations to BCVA, choroidal parameters exhibited no predictive impact for BCVA in multiple regression analysis. Indeed, visual outcome has been proven to be determined by retinal morphology and functioning more directly (2, 43), but accompanying changes in the choroid may drive or facilitate the process of retinal deterioration in iERMs (28, 31). Future prospective observations and additional clinicopathologic studies are needed to examine the microanatomy of the outer retina and choroid, thus fully understanding the sequence of events in the development of retinal and choroidal alterations in iERMs.

There were several limitations in our study. First, the total number of enrolled subjects was relatively small considering that there were four subgroups altogether, and the variance in sample size among the four groups, although inevitable, may induce statistical unreliability regarding the study conclusion. Second, the observational nature of this study narrowed the generalization of our conclusions, and was also insufficient to explicate the precedence of retinal and choroidal changes during the progression of iERMs. Last, although many parameters were obtained using unbiased automated software, a few retinal biomarkers, including the thickness of inner retinal layer and outer retinal distortion were still manually assessed. Future robust tools are urgently needed to automatically segment and identify retinal and choroidal abnormalities.

In conclusion, this study complemented our present understanding of the posterior segment structural and vascular alterations in iERMs. CVI varied, while SFCT and CCP were comparable, among different stages of iERMs. Functional damage to retinal integrity was determined to be a strong contributor to visual acuity reduction in iERMs. Besides, the correlation of CVI to both inner and outer retinal morphologic biomarkers indicated that future studies with larger sample sizes and longitudinal observations are needed to fully examine the possible mechanisms of retinal and choroidal alterations in the development of iERMs.

## Data availability statement

The raw data supporting the conclusions of this article will be made available by the authors, without undue reservation.

## Ethics statement

The studies involving human participants were reviewed and approved by the Institutional Review Board of Peking University Third Hospital. Written informed consent for participation was not required for this study in accordance with the national legislation and the institutional requirements.

## Author contributions

CW and XL: conceptualization. XL: data curation and funding acquisition. XW: formal analysis, investigation, and writing—original draft. XW and JY: methodology. CW: supervision. JY: writing—review and editing. All authors have read and agreed to the published version of the manuscript.
