# Supplementary material for: Choroidal morphologic and vascular features in patients with unilateral idiopathic epiretinal membranes: An optical coherence tomography analysis integrated with assessment of retinal layers
Source: Front Med (Lausanne). 2023 Jan 6;9:1083601. doi: 10.3389/fmed.2022.1083601 (PMC9853170; doi:10.3389/fmed.2022.1083601)
Supplement: Supplementary file 1 [file Image_1.pdf]

Supplementary Figure 1. Depictions of thickness measurement points and boundary of each retinal layer evaluated in eyes with stage 3 epiretinal membrane (A) and stage 4 epiretinal membrane (B). The underlying choroid in both images was displayed after binarization and identification for vascular and stromal components in a 2-mm wide region.

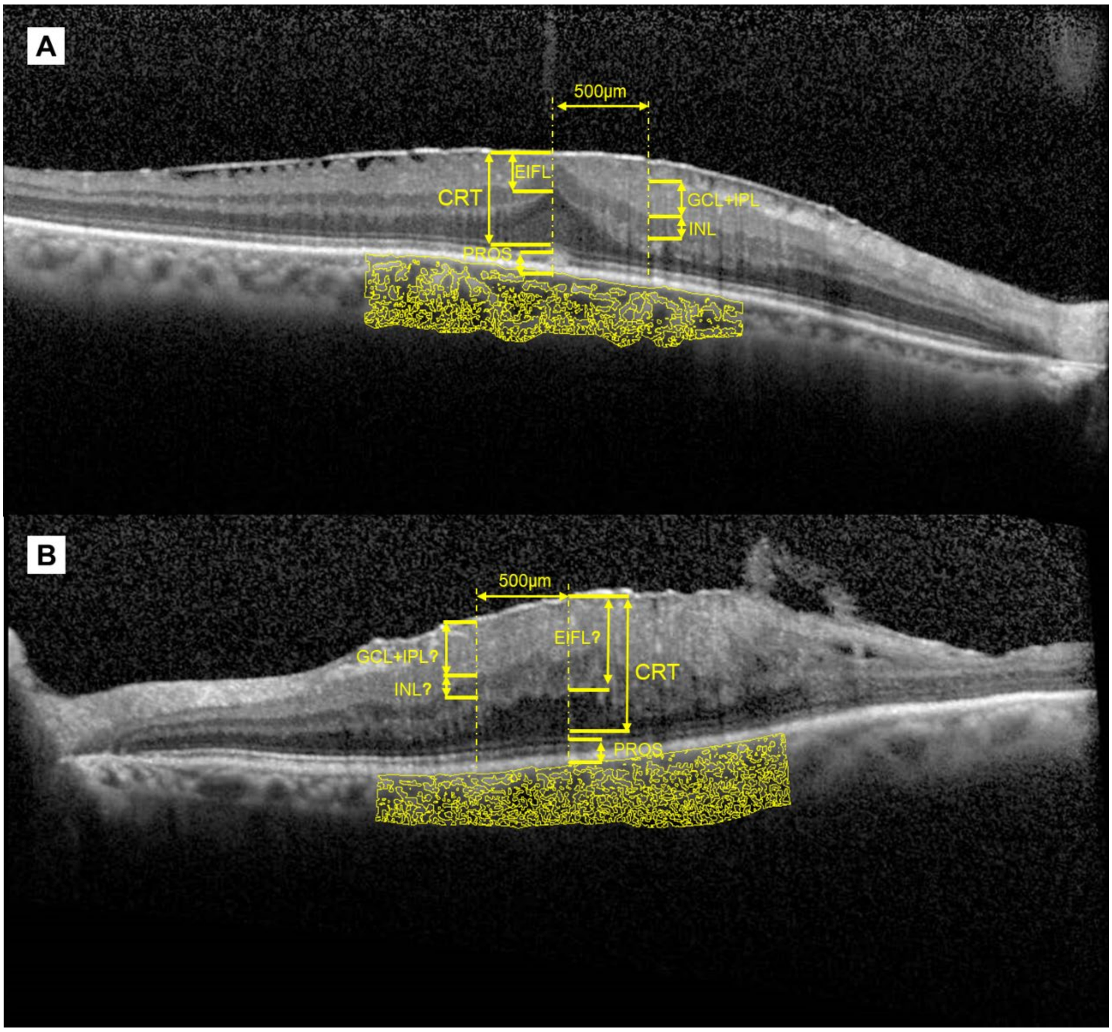

Measured at fovea: 1) CRT, central retinal thickness, from the external limiting membrane to the internal limiting membrane; 2) EIFL, ectopic inner foveal layers, from the upper limit of the outer nuclear layer to the internal limiting membrane; and 3) PROS, photoreceptor outer segment, from the inner border of the ellipsoid zone to the inner surface of the retinal pigment epithelium.

Measured at 500μm nasal to the fovea: 1) GCL + IPL, ganglion cell layer and inner plexiform layer; and 2) INL, inner nuclear layer.

Assessment of EIFL, GCL+IPL and INL was relinquished in stage 4 iERMs for the disorganization of retinal layers in advanced iERMs.
