# Supplementary material for: Choroidal morphologic and vascular features in patients with unilateral idiopathic epiretinal membranes: An optical coherence tomography analysis integrated with assessment of retinal layers
Source: Front Med (Lausanne). 2023 Jan 6;9:1083601. doi: 10.3389/fmed.2022.1083601 (PMC9853170; doi:10.3389/fmed.2022.1083601)
Supplement: Supplementary file 2 [file Table_1.docx]

**Supplementary Table 1.** Exclusion criteria in the screening of eligible subjects.

| **Exclusion criteria** |
| --- |
| - Any previous intraocular surgery with the exclusion of uncomplicated phacoemulsification |
| - History of retinal detachment |
| - Intermediate or advanced age-related macular degeneration |
| - History of choroidal neovascularization of any etiology |
| - Central serous chorioretinopathy |
| - Proliferative diabetic retinopathy |
| - Nonproliferative diabetic retinopathy with history of clinically significant diabetic macular edema |
| - Macular teleangectasias |
| - Tractional and degenerative lamellar macular holes |
| - History of central or branch retinal vein occlusion and central or branch retinal artery occlusion |
| - Advanced glaucoma, or optic neuropathy of any kind |
| - History of inflammatory eye disorders |
| - History of Irvine-Gass syndrome |
| - History of endophthalmitis or any other intraocular infection |
| - Retinal dystrophies |
| - Foveal hypoplasia/fovea plana |
| - History of ocular trauma |
| - Any other potential retinochoroidal cause of vision loss other than epiretinal membranes |
| - Uncontrolled hypertension (defined as office BP ≥140/90 mm Hg) ^a^ |
