# Supplementary material for: Choroidal morphologic and vascular features in patients with unilateral idiopathic epiretinal membranes: An optical coherence tomography analysis integrated with assessment of retinal layers
Source: Front Med (Lausanne). 2023 Jan 6;9:1083601. doi: 10.3389/fmed.2022.1083601 (PMC9853170; doi:10.3389/fmed.2022.1083601)
Supplement: Supplementary file 3 [file Table_2.docx]

**Supplementary Table 2**. Measured locations and boundaries for all retinal layers evaluated in the study.

| **Retinal layer** | **Measured points** | **Boundary** | **References** |
| --- | --- | --- | --- |
| **CRT**, central retinal thickness | Subfoveal | From the external limiting membrane to the internal limiting membrane | [1-4] |
| **GCL+IPL**, ganglion cell layer and inner plexiform layer | 500 μm from the foveal center, the point closest to the fovea in which all retinal layer thicknesses were clearly measurable | From the upper limit of the outer nuclear layer to the internal limiting membrane | [5,6] |
| **INL**, inner nuclear layer | 500 μm from the foveal center | From the outer surface of the inner plexiform layer to the inner surface of the outer plexiform layer | [5] |
| **EIFL**, ectopic inner foveal layers | Subfoveal | From the upper limit of the outer nuclear layer to the internal limiting membrane | [1,7] |
| **PROS**, photoreceptor outer segment |  | From the inner border of the ellipsoid zone to the inner surface of the retinal pigment epithelium | [8-11] |

**References**

[1]. Govetto A, Lalane RA, Sarraf D, Figueroa MS, Hubschman JP. Insights Into Epiretinal Membranes: Presence of Ectopic Inner Foveal Layers and a New Optical Coherence Tomography Staging Scheme. Am J Ophthalmol. 2017;175:99-113.

[2]. Shimozono M, Oishi A, Hata M, Matsuki T, Ito S, Ishida K, et al. The significance of cone outer segment tips as a prognostic factor in epiretinal membrane surgery. Am J Ophthalmol. 2012;153(4):698-704.

[3]. Watanabe K, Tsunoda K, Mizuno Y, Akiyama K, Noda T. Outer retinal morphology and visual function in patients with idiopathic epiretinal membrane. JAMA Ophthalmol. 2013;131(2):172-7.

[4]. Suh MH, Seo JM, Park KH, Yu HG. Associations between macular findings by optical coherence tomography and visual outcomes after epiretinal membrane removal. Am J Ophthalmol. 2009;147(3).

[5]. Kim JH, Kang SW, Kong MG, Ha HS. Assessment of retinal layers and visual rehabilitation after epiretinal membrane removal. Graefes Arch Clin Exp Ophthalmol. 2013;251(4):1055-64.

[6]. Lee EK, Yu HG. Ganglion cell-inner plexiform layer thickness after epiretinal membrane surgery: a spectral-domain optical coherence tomography study. Ophthalmology. 2014;121(8):1579-87.

[7]. Doguizi S, Sekeroglu MA, Ozkoyuncu D, Omay AE, Yilmazbas P. Clinical significance of ectopic inner foveal layers in patients with idiopathic epiretinal membranes. Eye (Lond). 2018;32:1652-60.

[8]. Shiono A, Kogo J, Klose G, Takeda H, Ueno H, Tokuda N, et al. Photoreceptor outer segment length: a prognostic factor for idiopathic epiretinal membrane surgery. Ophthalmology. 2013;120(4):788-94.

[9]. Hashimoto Y, Saito W, Saito M, Hirooka K, Fujiya A, Yoshizawa C, et al. Retinal outer layer thickness increases after vitrectomy for epiretinal membrane, and visual improvement positively correlates with photoreceptor outer segment length. Graefes Arch Clin Exp Ophthalmol. 2014;252(2):219-26.

[10]. Yüksel K, Karaküçük Y, Özkaya A, Pekel G, Baz Ö, Alagöz C, et al. Comparison of photoreceptor outer segment length in diabetic and idiopathic epiretinal membranes. Eye (Lond). 2015;29(11):1446-52.

[11]. Kinoshita T, Imaizumi H, Miyamoto H, Katome T, Semba K, Mitamura Y. Two-year results of metamorphopsia, visual acuity, and optical coherence tomographic parameters after epiretinal membrane surgery. Graefes Arch Clin Exp Ophthalmol. 2016;254(6):1041-9.
